# Alleviation of dry mouth by saliva substitutes improved swallowing ability and clinical nutritional status of post-radiotherapy head and neck cancer patients: a randomized controlled trial

**DOI:** 10.1007/s00520-019-05132-1

**Published:** 2019-11-15

**Authors:** Sumalee Nuchit, Aroonwan Lam-ubol, Wannaporn Paemuang, Sineepat Talungchit, Orapin Chokchaitam, On-ong Mungkung, Tippawan Pongcharoen, Dunyaporn Trachootham

**Affiliations:** 1 Master of Science Program in Nutrition and Dietetics, Institute of Nutrition, Mahidol University, Nakhon Pathom, Thailand; 2 Faculty of Dentistry, Srinakarinwirot University, Bangkok, Thailand; 3 Department of Vascular Surgery, Faculty of Medicine Siriraj Hospital, Mahidol University, Bangkok, Thailand; 4 Department of Dental Service, Chonburi Cancer Hospital, Chonburi, Thailand; 5 Institute of Nutrition, Mahidol University, 999 Phutthamonthon Sai 4 Road, Salaya, Nakhon Pathom, 73170 Thailand

**Keywords:** Dysphagia, Xerostomia, Saliva substitute, Nutritional status, Head and neck cancer, Radiation therapy

## Abstract

**Purpose:**

The aim of this study is to investigate the effect of an edible saliva substitute, oral moisturizing jelly (OMJ), and a topical saliva gel (GC) on dry mouth, swallowing ability, and nutritional status in post-radiotherapy head and neck cancer patients.

**Methods:**

Sixty-two post-radiation head and neck cancer patients with xerostomia completed a blinded randomized controlled trial. They were advised to swallow OMJ (*n* = 31) or apply GC orally (*n* = 31) for 2 months. Outcome measures were assessed at baseline, 1, and 2 months, including subjective and objective dry mouth (Challcombe) scores, subjective swallowing problem scores (EAT-10), water swallowing time, clinical nutritional status (PG-SGA), body weight, and dietary intake.

**Results:**

After 1 and 2 months of interventions, subjective and objective dry mouth scores, subjective swallowing problem scores, swallowing times, and clinical nutritional status in both groups were significantly improved (*p* < 0.0001). Compared to GC, OMJ group had higher percent improvement in all outcome measures (*p* < 0.001) except swallowing time and clinical nutritional status. Interestingly, subjective dry mouth scores were significantly correlated with subjective swallowing problem scores (*r* = 0.5321, *p* < 0.0001).

**Conclusions:**

Continuous uses of saliva substitutes (OMJ or GC) for at least a month improved signs and symptoms of dry mouth and enhanced swallowing ability. An edible saliva substitute was superior to a topical saliva gel for alleviating dry mouth and swallow problems. These lead to improved clinical nutritional status. Thus, palliation of dry mouth may be critical to support nutrition of post-radiotherapy head and neck cancer patients.

**Clinical trial registry:**

Clinicaltrials.gov NCT03035825

**Electronic supplementary material:**

The online version of this article (10.1007/s00520-019-05132-1) contains supplementary material, which is available to authorized users.

## Introduction

Saliva plays a critical role in chewing, swallowing, and taste perception [1]. Therefore, a decrease in the quantity and quality of saliva (hyposalivation) could result in swallowing difficulty (dysphagia), reduced intake, and consequently malnutrition [2]. Complaint of dry mouth (xerostomia) is common in patients with radiation-induced hyposalivation [2]. Head and neck cancer patients received radiotherapy exceeding 60 Grey (Gy) often had xerostomia together with dysphagia even after the cancer is remitted [3, 4]. These complications influence the patient’s appetite, food preference, and dietary intake. Consequently, these could lead to inadequate energy and nutrient intake causing malnutrition [5, 6]. In fact, a previous study showed that xerostomia was the most important factor associated with weight loss in cancer survivors after completion of radiotherapy [7]. Malnutrition in cancer patients has a great impact on the treatment outcome, length of hospital stays, quality of life, morbidity, and mortality [8]. Therefore, it has been proposed that effective management of dry mouth symptoms may improve nutritional status of the patients [9]. Currently, no research supports such hypothesis.

Current management of dry mouth symptoms includes sipping water frequently, taking medication to stimulate the production of saliva, or using saliva substitute to increase the moisture in the mouth [4, 10]. Systemic reviews concluded that oral mucosal lubricant (artificial saliva or so called “saliva substitute”) was one of the most effective intervention to alleviate dry mouth in cancer patients who have undergone radiation therapy [10, 11]. Nevertheless, there were no reports regarding the effect of saliva substitute on swallowing ability and nutritional status. Because of preservatives use, commercially available saliva substitutes are not recommended to be swallowed [12]. Furthermore, their side effects especially allergic reactions (rash; swelling of the mouth, face, lips, or tongue) have been reported [12]. Recently, Dental Innovation Foundation under Royal Patronage (DIF), a non-profit organization in Thailand, had developed a novel edible saliva substitute called oral moisturizing jelly (OMJ). The product can be swallowed from oral cavity through oropharynx which resembles natural saliva. A previous study in xerostomic elderly patients with hypertension and diabetes mellitus showed that continuous intake of OMJ for 4 weeks significantly reduced signs and symptoms of dry mouth, and achieved more than 80% satisfaction [13]. Recently, DIF have produced a new version of OMJ. It was manufactured by ultra-high temperature (UHT) processing for longer term storage and packed in smaller cup for convenience to consumption. Even though OMJ has been proven to be effective in reducing dry mouth symptoms, its effect on swallowing ability and nutrition has never been explored.

This study was aimed to investigate the effect of an edible saliva substitute OMJ on dry mouth, swallowing ability, and nutritional status of post-radiation head and neck cancer patients with xerostomia, in comparison with that of a commercially available saliva gel (GC) giving topically.

## Methods

The protocol of this work can be accessed at https://clinicaltrials.gov/ct2/show/NCT03035825?term=NCT03035825&rank=1

### Ethics

This study had been approved by the Ethics Committee of Chonburi Cancer Hospital (COA. No. 7/2016), the Mahidol University Central Institutional Review Board (Protocol No. MU-CIRB 2017/163.0809, COA. No. MU-CIRB 2017/165.0811), and the Ethics coammittee of Faculty of Dentistry, Srinakharinwirot University (COA. No. DENTSWU-EC26/2560).

### Study design, blinding, random allocation, and concealment

This study was a single-blinded randomized controlled trial. Participants were randomly allocated into two groups (1:1), i.e., OMJ group and GC groups. The participants were randomized to each group using minimization by matching age, sex, baseline subjective dry mouth score, and body mass index (BMI). Participants earlier recruited into the study were randomly assigned into two groups. Then, participants later recruited were assigned to minimize the difference in those factors between groups. Although the products were given to participants with no labels, packaging of two products were different; i.e., cup for OMJ and tube for GC. Therefore, some participants could recognize the products. Nevertheless, all data collectors and statistical analyzer were blinded. The product manufacturer and a researcher who functioned as random assigner were not involved in data collection and statistical analysis.

### Study site and participants

The participants were recruited from three sites including the dental department of the Chonburi Cancer Hospital, the Institute of Nutrition, Mahidol University, and Faculty of Dentistry, Srinakharinwirot University. Inclusion criteria were being diagnosed with head and neck cancer, being 30–70 years old, having completed radiation therapy at least 1 month, having dry mouth with subjective dry mouth score ≥ 3, having completed chemotherapy at least 2 weeks (if applicable), being able to eat by mouth, having no high risks of choking, being able to understand Thai language, and sign a written consent by themselves. Exclusion criteria were having inflammation of oral mucosa (mucositis grade ≥ 1), having oral infection, such as candidiasis, choking when consuming the products, and having cancer recurrence. All participants signed their written informed consents prior to data collection. Their identities were protected, following guidelines of the International Conference on Harmonization Good Clinical Practice (ICH-GCP).

### Sample size and power

The main objective of this work was to compare percent baseline of subjective dry mouth scores after 2 months of interventions between two groups. Thus, the sample size was calculated using non-centrality parameter of unpaired *t* test. Mean and SD from pilot data of 30 post-radiotherapy cancer patients (*n* = 15 each group) were used to obtain the effect size of 0.8. Level of significance was set as 0.05, power of 0.8. The calculated total sample size was 60 (at least 30 each group). To account for possible 15% drop-out, at least 70 participants needed to be recruited (35 each group).

### Intervention and materials

Participants in the study group were instructed to daily swallow 1–2 teaspoons of OMJ six times per day (every 3 h) for 2 months. Participants in the control group were instructed to topically apply a pea-sized drop of GC dry mouth gel to oral cavity six times per day (every 3 h) for 2 months as well. OMJ was obtained from the Dental Innovation Foundation under Royal Patronage (DIF), a non-profit organization. OMJ contains water, monosodium phosphate, disodium phosphate, carboxymethylcellulose, gelatin, glycerol, xanthan gum, and strawberry flavor. GC dry mouth gel was purchased from the GC Dental Products Corporation Company, Japan. GC contains water, sodium citrate, sodium carboxymethylcellulose, carrageenan, polyglycerol and xanthan gum, yoghurt flavor, limonen, linalool, citral, benzyl alcohol, and ethyl p-hydroxybenzoate. Both OMJ and GC contain zero calories.

### Study procedures

The study was performed according to the Declaration of Helsinki and ICH-GCP. All participants were randomized with minimization by age, sex, subjective dry mouth score, and BMI into two groups, i.e., study group (OMJ) and control group (GC). The outcome measures were evaluated at 0, 1, and 2 months after interventions. The primary outcome measure included subjective dry mouth scores. The secondary outcome measures comprised objective dry mouth scores, subjective swallowing problem score, water-swallowing time, clinical nutritional status, energy intake, and body weight. Throughout the study, all participants were asked to daily record their use of product in the subject diaries to ensure adherence to the intervention protocol. Any adverse events such as nausea, vomiting, diarrhea, swollen lips, and a rash were recorded.

### Outcomes

#### Subjective dry mouth score

A validated questionnaire was used to assess dry mouth symptoms as previously described [13]. There are six questions for symptoms of dry mouth, oral discomfort, awake at night to drink water, speech problem, swallowing difficulty, and ill-fitting dentures. Each participant described the magnitude of each problems in visual analog scale between 0 and 10 (no problems = 0, extremely troublesome = 10). The average of all scores (sum of total scores divided by number of questions) was used for interpretation. Score ≥ 3 indicates dry mouth symptoms.

#### Objective dry mouth score

Oral examination by a dentist specialized in oral medicine was performed to determine signs of dry mouth by using Challacombe Scale [14]. Details of scale and scoring were described in Electronic supplementary material.

#### Subjective swallowing problem score

A validated Eating Assessment Tool (EAT-10) was used to evaluate the degree of each swallowing problem [15]. Details of the EAT-10 questions and scoring were described in Electronic supplementary material.

#### Objective swallowing ability score

Water swallowing test (WST) was used to evaluate swallowing ability [16]. Each participant was asked to swallow 30 ml of room temperature water and the swallowing time was recorded. Normal swallowing time for adult age below 60 is between 0 and 5 s, while that of elderly age above 60 is between 0 and 7 s. Swallowing time longer than the normal range suggests abnormal swallowing [16].

#### Clinical nutritional status

Patient Generated-Subjective Global Assessment (PG-SGA) was used to evaluate nutritional status of cancer patients as described [17]. It is divided into two components, i.e., the medical history and the physical examination. The presence of nutrition-related signs and symptoms and short-term weight loss. Nutrition status was categorized as A (well-nourished), B (moderately malnourished), and C (severely malnourished). Nutrition triage scores were categorized to needs for nutrition therapy as (0-1) no interventions required, (2-3) patients and family education required, (4-8) interventions by dietitians required, and (≥ 9) nutrition therapy critically needed [17].

#### Body weight and body mass index

Body weight was measured by using a body composition monitor machine (TANITA BC-730, Tanita Corporation, Tokyo, Japan). Body mass index was calculated from body weight/height^2^. Height was measured by using height meter.

#### Energy intake

Three-day food record was used to evaluate energy intake as described [18]. The participants recorded all foods and beverages consumed since wake up to sleep for 3 days consisting of two weekdays and one weekend day. To improve accuracy of the data, additional information such as ready-to-eat food, made-to-order, or home-made were provided by the participants. The average energy intake for each visit of each participant was calculated by using INMUCAL-Nutrients Version 3. The program can analyze nutritive content of foods based on the Thai food composition database [19, 20].

### Statistics

Since the complete data from 62 participants provided adequate power of over 0.9, per protocol analysis was used with no needs for imputation. All statistical analyses were performed by a researcher who was blinded to randomization. Graphing and statistical analyses were performed using GraphPad Prism v. 7. Power analysis and sample size calculation were performed using G-power v.3.0.10. A significance level of 5% (*p* < 0.05) was used for all analyses. Normality of data distribution was assessed by D’Agostino and Pearson normality test. Participant characteristics with numerical scale were compared between groups by using unpaired *t* test. Comparison of baseline categorical data was analyzed by using Fisher’s exact test and Chi-square test as specified. Repeated measure ANOVA with Greenhouse-Geisser correction followed by Tukey’s multiple comparison test was used to compare changes in numerical outcome measures among 0, 1, and 2 months in the same group. Changes in objective dry mouth scores, subjective swallowing problem scores, and swallowing times after intervention were compared and categorized as same (similar scores to baseline), better (lower scores than baseline), or worse outcomes (higher scores than baseline). Comparison of outcome measures between OMJ and GC groups at the same time point was analyzed by using unpaired *t* test and chi-square test for numerical and categorical data, respectively. Correlation between subjective dry mouth score and subjective swallowing difficulty score were analyzed by using Pearson correlation analysis.

Treatment fidelity was described in electronic supplementary material.

## Results

### Participant flow chart

**Fig. 1:**
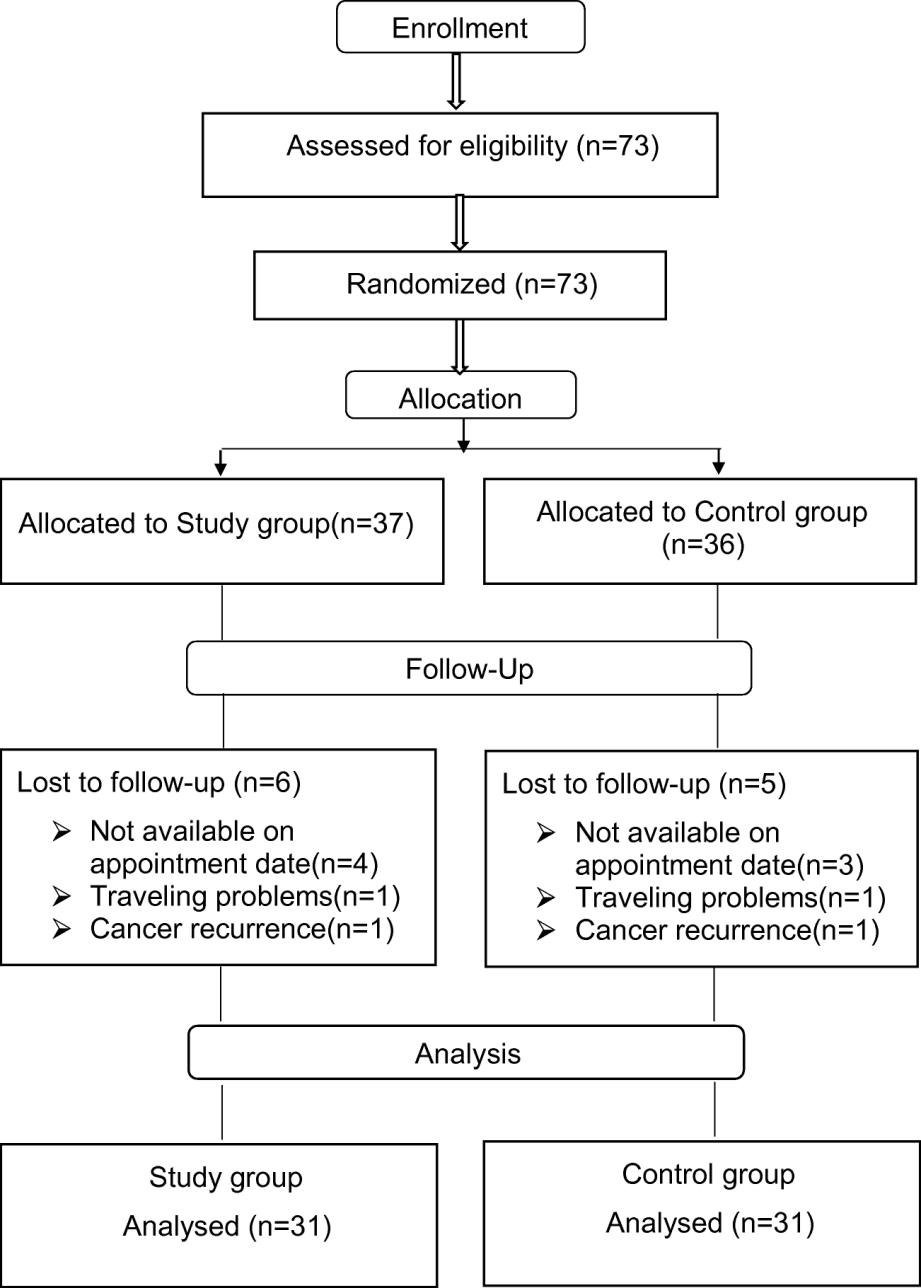
Participants’ flow chart. The diagram depicts the number of recruited volunteer and actual number of participants included in data analysis

The duration of data collection in this study was from November 2017 to November 2018. As shown in the participant flow chart (Fig. 1), there were 73 eligible participants. However, 11 participants were lost to follow-up. Sixty-two participants completed the study (85% of original participants) and were included in the analysis (*n* = 31 each group).

### Baseline characteristics

Table 1 showed that all baseline characteristics of the analyzed participants in study (OMJ) and control (GC) groups were not statistically different (*p* > 0.05). All participants were post-radiotherapy head and neck cancer survivors with complete remission. Average duration after radiotherapy of the participants was 28.4 ± 38.7 months. Most participants received conventional radiation therapy for treatment of nasopharyngeal, tongue, and oral cavity cancer; therefore, the radiation fields covered all salivary glands. Thirty-two percent and 22% of participants in in OMJ and GC groups, respectively, were treated with intensity modulated radiotherapy (IMRT) or volumetric modulated arc therapy (VMAT). IMRT and VMAT had been shown to reduce radiation doses for parotid but not submandibular salivary gland [21]. Thus, xerostomia problem of this subgroup may be less than the conventional one.

**Table 1:** Baseline characteristics of participants

Characteristics	Control group (GC) (*n* = 31)	Study group (OMJ) (*n* = 31)	*P* value
Age, mean ± SD (year)	55.5 ± 10.5	55.5 ± 9.3	0.9898^a^
Sex, *n* (%)			0.7863^b^
- Male - Female	20 (64.5) 11 (35.5)	22 (71.0) 9 (29.0)	
Subjective dry mouth score, mean ± SD	5.2 ± 1.5	5.6 ± 1.3	0.3355^a^
Body mass index, mean ± SD (kg/m^2^)	22.1 ± 4.1	20.8 ± 3.6	0.1894^a^
Body weight, mean ± SD (kg)	59.6 ± 13.2	55.7 ± 13.5	0.2548^a^
Baseline energy intake, mean ± SD (Kcal)	1465 ± 455	1579 ± 440	0.3923^a^
Number of radiations, mean ± SD (fractions)	33 ± 3.1	33 ± 2.4	0.6872^a^
Radiation dose, mean ± SD (Gy)	6600 ± 620	6600 ± 480	0.6872 ^a^
Duration after radiotherapy, mean ± SD (month)	28.4 ± 38.7	24.9 ± 25.3	0.6699^a^
Type of radiation, *n* (%)			0.205^c^
- Cobalt-60, LINAC -IMRT, VMAT (parotid gland sparing)	21 (67.8) 10 (32.2)	24 (77.4) 7 (22.6)	
Diagnosis of disease status Complete remission	31 (100)	31 (100)	> 0.99
Clinical staging prior to radiotherapy			0.335
Stage 1-2 Stage 3-4	4 (12.9) 27 (87.1)	6 (19.4) 25 (80.6)	
Location of cancer history, *n* (%)			0.9957 ^c^
- Nasopharynx - Tongue - Oral cavity - Larynx - Other	11 (35.5) 5 (16.1) 9 (29) 3 (9.7) 3 (9.7)	10 (32.3) 5 (16.1) 9 (29) 3 (9.7) 4 (12.9)	

p value (s) were from ^a^ unpaired *t* test, ^b^ Fisher exact test, and ^c^ Chi-square test; number in parentheses indicated percent of total participants

*IMRT* intensity modulated radiotherapy, *VMAT* volumetric modulated arc therapy, *LINAC* linear particle accelerator

### Effect of saliva substitutes on subjective and objective dry mouth scores

**Fig. 2:**
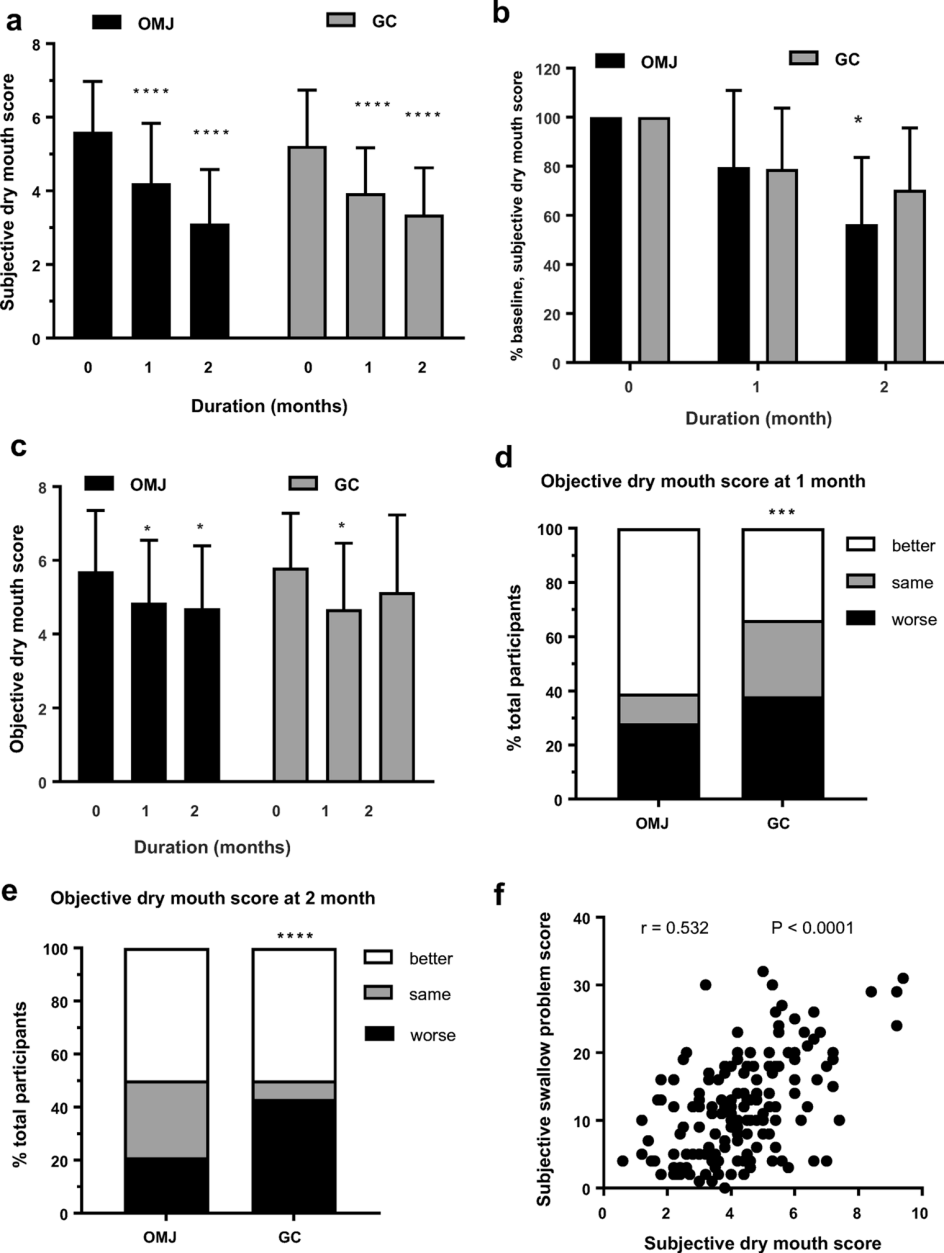
Effect of saliva substitutes on dry mouth. **a** Changes in subjective dry mouth scores (symptoms) in study group (OMJ; black bar) and control group (GC; gray bar) at baseline, after 1 and 2 months of interventions. Each bar represented mean ± SD of subjective dry mouth scores. (****) indicated *p* value < 0.0001; repeated measure ANOVA followed by Tukey’s multiple comparison test. **b** Comparison of changes in subjective dry mouth scores between study group (OMJ) and control group (GC) after 1 and 2 months of interventions. Each bar represented mean ± SD of percent baseline of subjective dry mouth scores. (*) indicated *p* value < 0.05; unpaired *t* test. **c** Changes in objective dry mouth scores (signs) in study group (OMJ; black bar) and control group (GC; gray bar) at baseline, after 1 and 2 months of interventions. Each bar represented mean ± SD of objective dry mouth scores. (*) indicated *p* value < 0.05; repeated measure ANOVA followed by Tukey’s multiple comparison test. **d**, **e** Comparison of changes in objective dry mouth scores between study group (OMJ) and control group (GC) after 1 month (**d**) or 2 months (**e**) of interventions. Stacked bar represented percent of participants with better, same or worse outcome, compared to their own baseline values. (***) indicated *p* value < 0.001, (****) indicated *p* value < 0.0001; Chi-square test. **f** Correlation between subjective dry mouth scores and subjective swallow problem scores. Each dot in the dot plot represented co-ordinates of subjective dry mouth scores and subjective swallow problem scores in the same participants. (*r* = 0.532, *p* < 0.0001; Pearson correlation analysis)

As shown in Fig. 2a, subjective dry mouth scores in both OMJ and GC groups were significantly decreased after 1 and 2 months of interventions in time-dependent manner (*p* < 0.0001). Interestingly, Fig. 2b showed that percent baseline of subjective dry mouth scores in OMJ group at 2 months was significantly less than that of GC group (*p* < 0.05). The data suggested superior effect of OMJ in relieving symptoms of dry mouth. As shown in Fig. 2c, objective dry mouth scores in the OMJ group were significantly decreased after 1 and 2 months of intervention (*p* < 0.05). In contrast, those scores in GC group were significantly decreased only at 1 month (*p* < 0.05) but resumed at 2 months after intervention. Consistently, Fig. 2d, e showed that OMJ group had significantly higher percent of improved objective dry mouth scores at 1 month and less percent of worse scores at 2 months, than those of GC group. The data implied better effect of OMJ in relieving signs of dry mouth.

### Correlation between subjective dry mouth and swallowing problem scores

Figure 2f illustrated significantly positive correlation between subjective dry mouth scores and subjective swallowing problem score (*r* = 0.5321, *p* < 0.0001). The data suggested that symptoms of dry mouth were associated with swallowing difficulty. And reduction in dry mouth scores could be linked with a decrease in swallowing problem.

### Effect of saliva substitutes on subjective and objective swallowing ability

**Fig. 3:**
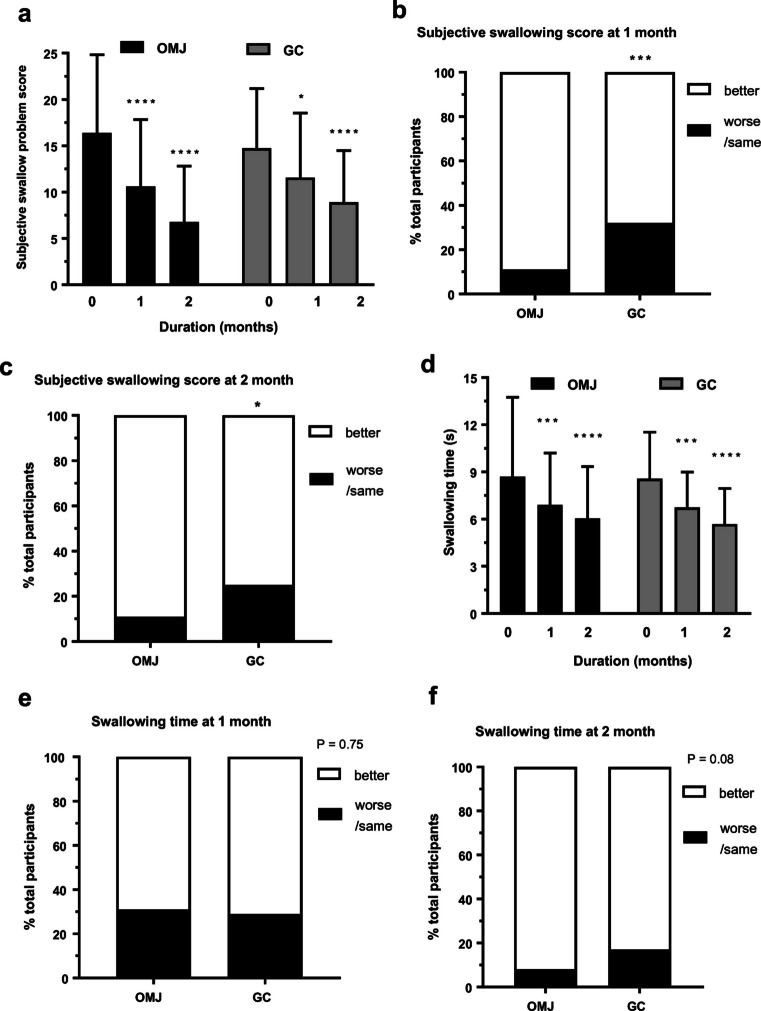
Effect of saliva substitutes on swallowing ability. **a** Changes in subjective swallowing problem scores (symptoms) in study group (OMJ; black bar) and control group (GC; gray bar) at baseline, after 1 and 2 months of interventions. Each bar represented mean ± SD of subjective swallowing problem scores. (****) indicated *p* value < 0.0001, (*) indicated *p* value < 0.05; repeated measure ANOVA followed by Tukey’s multiple comparison test. **b**, C Comparison of changes in subjective swallowing problem scores between study group (OMJ) and control group (GC) after 1 month (**b**) or 2 months (**c**) of interventions. Stacked bar represented percent of participants with better (white bar), same/worse (black bar) outcomes, compared to their own baseline values. (*) indicated *p* value < 0.05, (***) indicated *p* value < 0.001; Chi-square test. **d** Changes in swallowing time (objective swallowing ability) in study group (OMJ; black bar) and control group (GC; gray bar) at baseline, after 1 and 2 months of interventions. Each bar represented mean ± SD of swallowing times. (***) indicated *p* value < 0.001, (****) indicated *p* value < 0.0001; repeated measure ANOVA followed by Tukey’s multiple comparison test. **e**, **f** Comparison of changes in subjective swallowing problem scores between study group (OMJ) and control group (GC) after 1 month (**e**) or 2 months (**f**) of interventions. Stacked bar represented percent of participants with better (white bar), same/worse (black bar) outcomes, compared to their own baseline values. The indicated *p* values were from Chi-square tests

As shown in Fig. 3a, subjective swallowing problem scores in both OMJ and GC groups were significantly decreased after 1 and 2 months of interventions in time-dependent manner (*p* < 0.0001). Interestingly, Fig. 3b, c consistently showed that OMJ group had significantly higher percent of improved subjective swallowing scores at 1 and 2 months, than those of GC group (*p* < 0.001 and *p* < 0.05, respectively). The data indicated better effect of OMJ in relieving symptoms of swallowing difficulty. As shown in Fig. 3d, water swallowing time in both OMJ and GC groups were significantly decreased after 1 and 2 months of interventions in time-dependent manner (*p* < 0.0001). However, Fig. 3e, f showed no significant differences in percent improved swallowing time between groups at 1 month (*p* = 0.75) and 2 months (*p* = 0.08).

### Effect of saliva substitutes on nutritional status

**Fig. 4:**
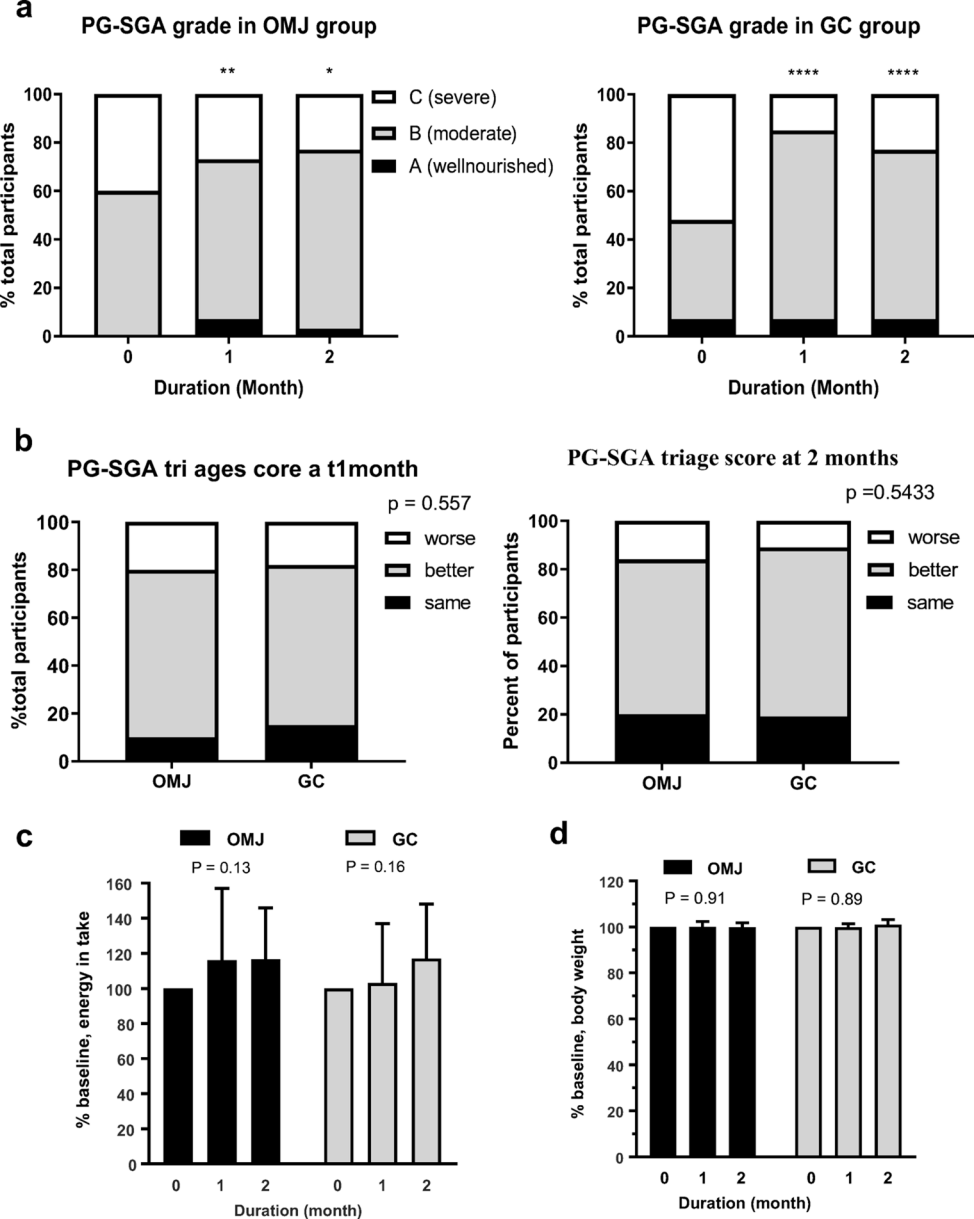
Effect of saliva substitutes on nutritional status. **a** Changes in PG-SGA categories of mild, moderate, and severe malnutrition in study group (OMJ; left panel) and control group (GC; right panel) after 1 and 2 months of interventions. Stacked bar represented percent of participants with severe (white bar), moderate (gray bar), and mild (black) malnutrition categories. (*), (**), (****) indicated *p* value < 0.05, 0.01, and 0.0001; Chi-square tests. **b** Comparison of changes in PG-SGA scores between study group (OMJ) and control group (GC) after 1 month (left panel) or 2 months (right panel) of interventions. Stacked bar represented percent of participants with better, same, or worse outcome, compared to their own baseline values. The indicated *p* values were from Chi-square tests. **c** Changes in energy intakes (% baseline) in study group (OMJ; black bar) and control group (GC; gray bar) at baseline, after 1 and 2 months of interventions. Each bar represented mean ± SD of percent baseline of energy intakes. The indicated *p* values were from repeated measure ANOVA. **d** Changes in body weights (% baseline)in study group (OMJ; black bar) and control group (GC; gray bar) at baseline, after 1 and 2 months of interventions. Each bar represented mean ± SD of percent baseline of body weights. The indicated *p* values were from repeated measure ANOVA

Figure 4a (left and right panel) and Figure S1-A showed significantly reduced percent of severe malnutrition and requirement of nutrition therapy but increased percent of well-nourished and moderate malnutrition after 1 and 2 months of OMJ or GC interventions. The data suggested that both OMJ and GC may improve clinical nutritional status. In Figure S1-B, the baseline nutritional status in OMJ group was significantly worse than GC group. Nevertheless, Fig. 4b showed no significant differences in percent improved clinical nutritional status between OMJ and GC groups at 1 month (*p* = 0.56) and 2 months (*p* = 0.54). When considering factor-specific PG-SGA scores, there were significant improvements in three factors including food intake, symptoms, activities, and function in both groups (electronic supplementary material Table S1). As shown in Fig. 4c and Figure S1-**C**, energy intake in both OMJ and GC groups slightly increased after 1 and 2 months of intervention. However, the changes were not statistically significant (*p* = 0.13 and *p* = 0.16, respectively). Likewise, the changes of body weight after intervention in both OMJ and GC groups were not statistically significant as shown in Fig. 4d (*p* = 0.89 and *p* = 0.91, respectively).

### Adverse events of saliva substitutes

As shown in Table 2, some participants in GC group had non-serious adverse events including mouth pain (10%) and mouth ulcer (7%). In contrast, no participants in OMJ group had any adverse events during 2 months use of intervention.

**Table 2:** Adverse events after uses of saliva substitutes

Symptoms	Control group (GC) (*n* = 31)	Study group (OMJ) (*n* = 31)	
Mouth pain	3 (10%)	0	
Mouth ulcer	2 (7%)	0	

## Discussion

To our knowledge, this is the first experimental evidence showing that alleviation of dry mouth by saliva substitutes could improve swallowing ability and clinical nutritional status. Furthermore, OMJ, a new edible saliva substitute, was more effective in relieving symptoms of dry mouth and improving swallowing ability than those of GC, a topically applied saliva gel. Dry mouth and dysphagia (swallowing difficulty) are found in 40–60% of elderly people in assisted living facilities and nursing homes [22, 23]. Moreover, 90% of post-radiation cancer survivors reported xerostomia and dysphagia associated with malnutrition [7]. Therefore, the findings of this study highlight the importance of dry mouth management as a critical part in nutritional therapy especially for cancer patients.

Subjective and objective swallowing ability were improved in both OMJ and GC groups after at least 1 month of intervention. Nevertheless, OMJ showed better results in improving subjective swallowing ability. The significant correlation between subjective dry mouth and subjective swallowing problem scores suggested that the improvement in swallowing function likely resulted from alleviation of dry mouth by saliva substitutes. Therefore, the superior effect of OMJ in improving subjective dry mouth may explain its better outcome in reducing subjective swallow problems. Since the participants were instructed to swallow OMJ like natural saliva, its bathing effect on oral and throat mucosal walls may lubricate bolus of food, thereby facilitate swallowing [24]. This study used EAT-10 and water swallowing test which are screening assays to determine swallowing ability. Future studies should include more objective tests such as fiberoptic endoscopic evaluation of swallowing (FEES) evaluated by professionals such as speech pathologists.

Clinical nutritional status (PG-SGA categories) was improved in both OMJ and GC groups after at least 1 month of intervention. Analyses of factor-specific PG-SGA scores revealed the impact of both saliva substitutes on food intake, symptoms, activities, and function. Since symptoms appeared to be the most improved factor, alleviation of dry mouth symptoms by using saliva substitutes may result in improved overall nutritional status. It is worth noting that recovery of nutritional status is not solely dependent on improved saliva condition and swallowing ability. Self-compliance and social support related to food preparation, enjoyment, ability to eat, drink, and swallow due to the impact of time and effort with swallowing and the associated burden, meeting nutritional requirements with oral and administration of enteral nutrition should be recommended along with the application of saliva substitutes.

Although the changes in energy intakes of both groups were not statistically significant, there was tendency of increase in time-dependent manner. Since the saliva substitutes contain no calories, the increased energy intake likely resulted from improved swallowing ability. Though the swallowing ability has improved after a few months of intervention, eating behavior and body weight may require longer time to be changed [25]. Future studies should increase the duration of interventions to 3 months or 6 months to observe changes in dietary intake and body weight. In addition, changes in type and texture of food after using edible saliva gel should be systemically assessed using established system such as National Dysphagia Diet (NDD) or International Dysphagia Diet Standard Initiatives (IDDSI) categories [26, 27].

Adverse events were observed only in the GC group but not in OMJ group. In this study, five flavors of GC dry mouth gels were available including lemon, orange, mint, raspberry, and fruit-salad. In contrast, OMJ had only one flavor of strawberry. In fact, a previous study reported that a flavoring agent peppermint was associated with burning mouth and oral ulceration [28]. Interestingly, we observed that all participants with adverse events were those who used mint flavored GC gel. Therefore, the mouth pain and mouth ulcer were likely results of allergic reaction to mint flavor. Taken together, mint flavor should not be used for saliva substitute products. Strawberry flavored product seemed to be non-allergenic and more suitable for patients with xerostomia.

Recovery of salivary glands after radiotherapy depends on radiation techniques, radiation doses, and time [29–31]. For conventional technique, a study showed that xerostomia did not change significantly during 5 years after radiotherapy [29]. However, another study found a slight increase of salivary flow rate 6 months after radiation and a significantly increase at 5 years after radiation [30]. Furthermore, a recent study in patients receiving IMRT suggested that if the mean doses of radiation on a parotid salivary gland were less than 26 Gy, complete recovery of pre-radiation salivary flow rates could be possible at 36 months after radiotherapy [31]. In this study, most participants received conventional therapy while some received IMRT and VMAT. The average duration after radiotherapy of participants was 28.4 ± 38.7 months. Thus, we cannot exclude the possibility that the observed changes on alleviation of dry mouth, swallowing, and nutritional status in this study may be partly from self-recovery. Future clinical trials using placebo control with completely no effects are warranted to confirm the efficacy of salivary substitutes on swallowing and nutritional status.

Major strengths of the present study are the randomized and blinded design, the use of same data collector of each outcome for all participants and all visits, and the good compliance and adherence (15% drop-out). Since the target population is head and neck cancer patients who already finished their radiation therapy for at least 1 month, most participants no longer have to visit the hospitals. Therefore, it was quite challenging to have them come for follow-up visits of this study. In fact, our previous pilot study in this cancer patient group had 50% drop-out rate. Therefore, to achieve ethical merit and good adherence, we designed the research by using a commercial saliva gel, GC as control group instead of no treatment or placebo gel. Since all participants received beneficial products, they were willing to come for all follow-up visits and the high adherence of 85% was achieved.

Nevertheless, there are limitations of this study. First, we advised the participant to swallow OMJ but apply GC topically in the mouth. Since the participant who used GC did not spit it out, they may swallow some GC gel. This may explain the equal effect between GC and OMJ to improve swallowing time. Therefore, future studies should consider other kinds of saliva substitutes which really cannot be swallowed such as mouth wash. Second, this study included all patients who had dry mouth problems regardless of the radiation dose they received. Nevertheless, we tried to balance the radiation dose in both groups to achieve equal average of 33 fractions of radiation (66 Gy). Future studies should include stratification of radiation dose and compare the effect of saliva substitutes in various doses of radiation. Third, this study had no placebo control. Thus, the effect of saliva substitutes cannot be completely distinguished from self-recovery effect. Last, other factors such as self-compliance and social support were not included in data collection and analysis. Since this is considered the first study exploring the effect of saliva substitutes on nutrition, further large-scale studies with better design to manage the above-mentioned confounders are warranted.

In conclusion, the findings from this study suggested that continuous uses of saliva substitutes (OMJ or GC) for at least a month improved signs and symptoms of dry mouth and enhanced swallowing ability. An edible saliva substitute, OMJ was superior to GC for alleviation of dry mouth and subjective swallow problems. These together may lead to improved clinical nutritional status in post-radiotherapy head and neck cancer patients. Thus, an edible saliva substitute could be an alternative intervention for resuming oral moisture and swallowing ability, and improving nutritional status in post radiotherapy head and neck cancer patients. Furthermore, this approach may have broad applications in elderly with dysphagia and dry mouth. However, further long-term clinical studies in larger scale are warranted to confirm the comprehensive benefit of edible saliva substitute.

## Electronic supplementary material

ESM 1 (DOCX 74 kb)

## Abbreviations

BIA: Bioelectrical impedance assessment
BMI: Body mass index
EAT-10: Eating Assessment Tool
OMJ: Oral moisturizing jelly
PG-SGA: Patient Generate-Subjective Global Assessment
WST: Water swallowing test
