# Correction to: Alleviation of dry mouth by saliva substitutes improved swallowing ability and clinical nutritional status of post-radiotherapy head and neck cancer patients: a randomized controlled trial

**DOI:** 10.1007/s00520-025-09912-w

**Published:** 2025-09-03

**Authors:** Sumalee Nuchit, Aroonwan Lam-ubol, Wannaporn Paemuang, Sineepat Talungchit, Orapin Chokchaitam, On-ong Mungkung, Tippawan Pongcharoen, Dunyaporn Trachootham

**Affiliations:** 1 Master of Science Program in Nutrition and Dietetics, Institute of Nutrition, Mahidol University, Nakhon Pathom, Thailand; 2 Faculty of Dentistry, Srinakarinwirot University, Bangkok, Thailand; 3 Department of Vascular Surgery, Faculty of Medicine Siriraj Hospital, Mahidol University, Bangkok, Thailand; 4 Department of Dental Service, Chonburi Cancer Hospital, Chonburi, Thailand; 5 Institute of Nutrition, Mahidol University, 999 Phutthamonthon Sai 4 Road, Salaya, Nakhon Pathom, 73170 Thailand

**Correction to: Supportive Care in Cancer (2020) 28:2817–2828**

10.1007/s00520-019-05132-1

"Page 2818: Section Methods, subsection "Ethics" stated that

"This study had been approved by the Ethics Committee of Chonburi Cancer Hospital (COA. No. 7/2016), the Mahidol University Central Institutional Review Board (COA. No. 2017/163.0809), and the Ethics committee of Faculty of Dentistry, Srinakharinwirot University (COA. No. DENTSWU-EC26/2560)."

It should be corrected to

"This study had been approved by the Ethics Committee of Chonburi Cancer Hospital (COA. No. 7/2016), the Mahidol University Central Institutional Review Board (Protocol No. MU-CIRB 2017/163.0809, COA. No. MU-CIRB 2017/165.0811), and the Ethics committee of Faculty of Dentistry, Srinakharinwirot University (COA. No. DENTSWU-EC26/2560)."

The original article is corrected.

