# Supplementary material for: Alleviation of dry mouth by saliva substitutes improved swallowing ability and clinical nutritional status of post-radiotherapy head and neck cancer patients: a randomized controlled trial
Source: Support Care Cancer. 2019 Nov 15;28(6):2817–28. doi: 10.1007/s00520-019-05132-1 (PMC7181446; doi:10.1007/s00520-019-05132-1)
Supplement: Supplementary file 1 — (DOCX 74 kb) [file 520_2019_5132_MOESM1_ESM.docx]

**Supplemental data**

**Alleviation of Dry Mouth by Saliva Substitutes Improved Swallowability and Clinical Nutritional Status of Post-Radiotherapy Head and Neck Cancer Patients: a Randomized Controlled Trial**

Sumalee Nuchit^a^, Aroonwan Lam-ubol^b^, Wannaporn Paemuang^c^, Sineepat Talungchit^b^, Orapin Chokchaitam^c^, On-ong Mungkung^c^, Tippawan Pongcharoen^d^, and Dunyaporn Trachootham^d,^ *

^a^ Master of Science Program in Nutrition and Dietetics, Institute of Nutrition, Mahidol University, Nakhon Pathom, Thailand

^b^ Faculty of Dentistry, Srinakarinwirot University, Bangkok Thailand

^c^ Department of Dental Service, Chonburi Cancer Hospital, Chonburi, Thailand

^d^ Institute of Nutrition, Mahidol University, Nakhon Pathom, Thailand

***Corresponding author:** dunyaporn.tra@mahidol.ac.th; dif.dunyaporn@gmail.com

**Methods**

**Objective dry mouth score**: Challacombe Scale

Oral examination by a dentist specialized in oral medicine was performed to determine signs of dry mouth by using: Challacombe Scale. Ten signs of dry mouth included positivity on mirror stick test to - buccal mucosa, - tongue, frothy saliva, no saliva pooling, generalized shortened tongue papillae, altered gingival architecture (i.e. smooth), glassy appearance of oral mucosa, especially palate, lobulated/ fissured tongue, more than 2 teeth with cavities in neck of tooth, and debris on palate or teeth. One positive sign was counted as 1 point. Total scores were interpreted for degree of dryness. Score ≥ 2 indicated significant mouth dryness.

**Subjective swallowing problem score**: Eating Assessment Tool (EAT-10)

A validated Eating Assessment Tool (EAT-10) was used to evaluate the degree of each swallowing problem. Ten problems included weight loss due to swallowing difficulty, interfering with ability to go out for meals, extra efforts needed in swallowing liquid, extra efforts needed in swallowing solid food, extra efforts needed in swallowing pills, painful swallowing, reduced pleasure of eating, food stuck in the throat, cough when eating, stressful swallowing. Each participant described the magnitude of each problems in scoring between 0-4 (no problem = 0, severe problem = 4). Summation of scores for all questions were used to interpret the degree of swallowing problems.

**Treatment fidelity**

To ensure treatment fidelity, treatment designs for saliva substitutes and outcome measures were reviewed and approved by a team of four professional dentists and two registered dietitians. To ensure the consistency of the protocol, the data was collected by the same team. Each outcome for all participants was collected on every visit by the same researcher. To ensure consistency of intervention delivery, the instruction for use of OMJ and GC was delivered by one researcher for all participants to. Among 73 patients enrolled in the study, 62 patients (85%) returned for complete follow-up and reported continuous use of products. Fifteen percent of patients lost to follow-up due to various reasons including unavailability on appointment date, traveling problems and cancer recurrence. The 15% drop-out rate should be included in sample size calculation of future studies.

**Table S1: Effect of saliva substitutes on factor-specific PG-SGA scores**

| **Domain** | **Control group (GC)**  **n = 31** | | | | **Study group (OMJ)**  **n = 31** | | | |
| --- | --- | --- | --- | --- | --- | --- | --- | --- |
|  | **Baseline** | **1**  **month** | **2**  **months** | **P-value** | **Baseline** | **1**  **month** | **2**  **months** | **P-value** |
| **1. Weight** | 0.08 ± 0.06 | 0.3 ± 0.12 | 0.08 ± 0.06 | 0.102 ^a^ | 0 ± 0 | 0.2 ± 0.1 | 0.2 ± 0.1 | 0.135 ^a^ |
| **2. Food intake** | 1.5 ± 0.19 | 1.4 ± 0.15 | 1.2 ± 0.12 | 0.042^*, a^ | 1.41 ± 0.14 | 1.24 ± 0.11 | 1.13 ± 0.08 | 0.015^*, b^ |
| **3. Symptoms** | 4.05 ± 0.43 | 2.81 ± 0.38 | 2.59 ± 0.26 | 0.031^*, a^ | 4.45 ± 0.33 | 3.27 ± 0.33 | 3.03 ± 0.38 | 0.001^***, b^ |
| **4.** **Activities**  **and function** | 0.13 ± 0.07 | 0 ± 0 | 0 ± 0 | 0.0498^*, a^ | 0.41 ± 0.11 | 0.2 ± 0.1 | 0.07 ± 0.05 | 0.003^**, a^ |
| **5.** **Disease and**  **its relation to requirements** | 1.22 ± 0.09 | 1.22 ± 0.09 | 1.22 ± 0.09 | 0.376 ^b^ | 1.14 ± 0.06 | 1.14 ± 0.06 | 1.07 ± 0.05 | 0.135 ^a^ |
| **6.** **Metabolic**  **demand** | 0.04 ± 0.04 | 0.04 ± 0.04 | 0 ± 0 | 0.606 ^a^ | 0 ± 0 | 0.10 ± 0.07 | 0.07 ± 0.05 | 0.367 ^a^ |
| **7.** **Physical**  **Exam** | 1.44 ± 0.2 | 1.44 ± 0.2 | 1.44 ± 0.2 | 0.374 ^b^ | 1.3 ± 0.16 | 1.3 ± 0.16 | 1.3 ± 0.16 | 0.374 ^b^ |

Mean ± SEM, P-value (s) were from comparative analyses between baseline, 1 and 2 months after uses of GC or OMJ by using ^a^ Friedman test, ^b^ repeated measures ANOVA, * means p < 0.05; ** means p < 0.01, *** means p ≤ 0.001

**Figure S1: Changes in PG-SGA nutrition triage categories and energy intake**

**A**: Changes in PG-SGA nutrition triage categories in study group (OMJ; left panel) and control group (GC; right panel) after 1 and 2 months of interventions. Stacked bar represented percent of participants with A (white bar), B (gray bar) and C (black) malnutrition categories. (**) and (****) indicated p < 0.01 and p < 0.0001, respectively, Chi-square tests. **B**: Baseline PG-SGA categories of nutrition status in OMJ and GC groups. Stacked bar represented percent of participants with A (white bar), B (gray bar) and C (black) malnutrition categories. (**) indicated p < 0.01, Chi-square tests. **C**: Changes in daily energy intakes in study and control groups. Each bar represented mean ± SD of energy intake (kcal). P-values were from repeated measure ANOVA.

**
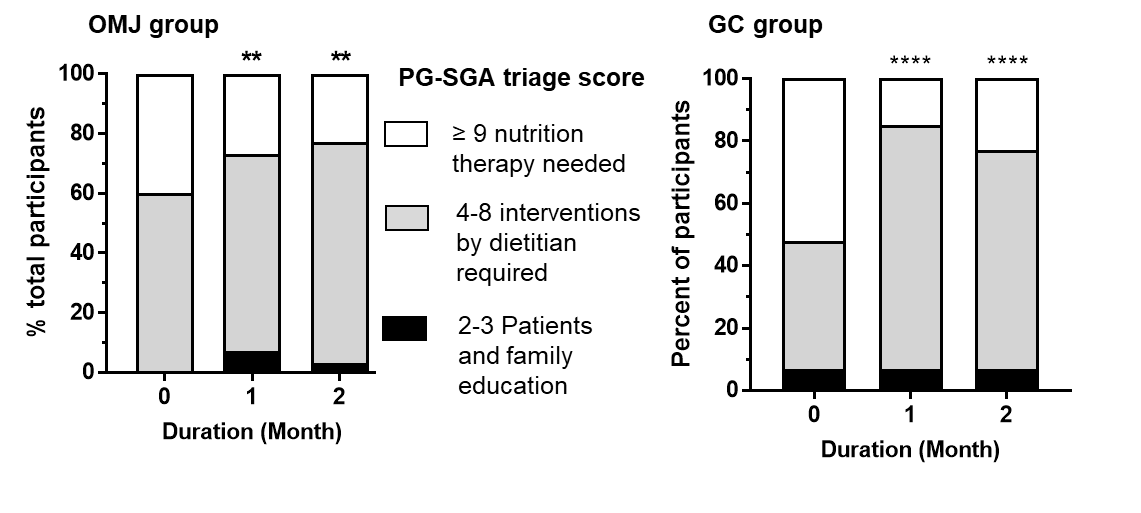
A**

**
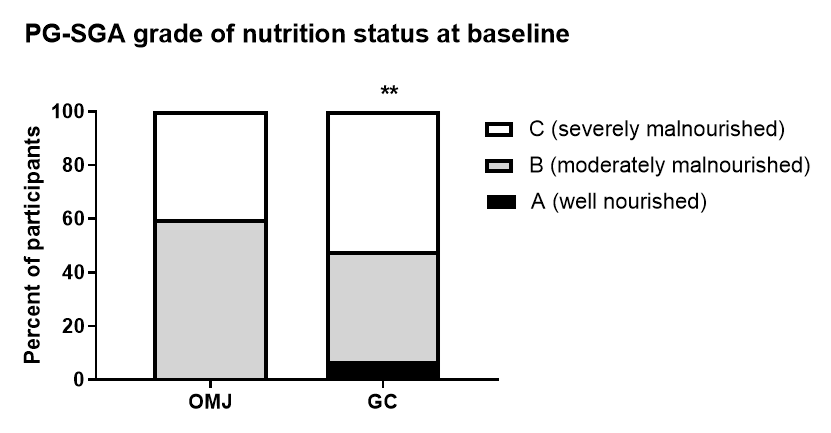
B**

**C**
